# Trends in malaria cases, hospital admissions and deaths following scale-up of anti-malarial interventions, 2000–2010, Rwanda

**DOI:** 10.1186/1475-2875-11-236

**Published:** 2012-07-23

**Authors:** Corine Karema, Maru W Aregawi, Alphonse Rukundo, Alain Kabayiza, Monique Mulindahabi, Ibrahima S Fall, Khoti Gausi, Ryan O Williams, Michael Lynch, Richard Cibulskis, Ngabo Fidele, Jean-Pierre Nyemazi, Daniel Ngamije, Irenee Umulisa, Robert Newman, Agnes Binagwaho

**Affiliations:** 1 Malaria and Other Parasitic Diseases Division (Rwanda Malaria Control Programme), Rwanda Biomedical Centre, Ministry of Health, Kigali, Rwanda; 2 World Health Organization, Global Malaria Programme, Geneva, Switzerland; 3 World Health Organization, Regional Office for Africa (AFRO), Brazzaville, Congo; 4 World Health Organization, Inter-Country Support Team for Southern and Eastern Africa, Regional Office for Africa (AFRO), Harare, Zimbabwe; 5 Single Project Management Unit-GF Projects-Ministry of Health-Rwanda, Kigali, Rwanda; 6 Maternal and Child Health Unit, Ministry of Health-Rwanda, Kigali, Rwanda; 7 Ministry of Health, Rwanda, Kigali, Rwanda; 8 Department of Global Health and Social Medicine, Harvard Medical School, Boston, USA; 9 Planning, M&E Coordination - Rwanda Biomedical Centre, Ministry of Health, Kigali, Rwanda

## Abstract

**Background:**

To control malaria, the Rwandan government and its partners distributed insecticide-treated nets (ITN) and made artemisinin-based combination therapy (ACT) widely available from 2005 onwards. The impact of these interventions on malaria cases, admissions and deaths was assessed using data from district hospitals and household surveys.

**Methods:**

District records of ITN and ACT distribution were reviewed. Malaria and non-malaria indictors in 30 district hospitals were ascertained from surveillance records. Trends in cases, admissions and deaths for 2000 to 2010 were assessed by segmented log-linear regression, adjusting the effect size for time trends during the pre-intervention period, 2000–2005. Changes were estimated by comparing trends in post-intervention (2006–2010) with that of pre-intervention (2000–2005) period. All-cause deaths in children under-five in household surveys of 2005 and 2010 were also reviewed to corroborate with the trends of deaths observed in hospitals.

**Results:**

The proportion of the population potentially protected by ITN increased from nearly zero in 2005 to 38% in 2006, and 76% in 2010; no major health facility stock-outs of ACT were recorded following their introduction in 2006. In district hospitals, after falling during 2006–2008, confirmed malaria cases increased in 2009 coinciding with decreased potential ITN coverage and declined again in 2010 following an ITN distribution campaign. For all age groups, from the pre-intervention period, microscopically confirmed cases declined by 72%, (95% Confidence Interval [CI], 12-91%) in 2010, slide positivity rate declined 58%, (CI, 47%–68%), malaria inpatient cases declined 76% (CI, 49%–88%); and malaria deaths declined 47% (CI, 47%–81%). In children below five years of age, malaria inpatients decreased 82% (CI, 61%-92%) and malaria hospital deaths decreased 77% (CI, 40%–91%). Concurrently, outpatient cases, admissions and deaths due to non-malaria diseases in all age groups either increased or remained unchanged. Rainfall and temperature remained favourable for malaria transmission. The annual all-cause mortality in children under-five in household surveys declined from 152 per 1,000 live births during 2001–2005, to 76 per 1,000 live births in 2006–2010 (55% decline). The five-year cumulative number of all-cause deaths in hospital declined 28% (8,051 to 5,801) during the same period.

**Conclusions:**

A greater than 50% decline in confirmed malaria cases, admissions and deaths at district hospitals in Rwanda since 2005 followed a marked increase in ITN coverage and use of ACT. The decline occurred among both children under-five and in those five years and above, while hospital utilization increased and suitable conditions for malaria transmission persisted. Declines in malaria indicators in children under 5 years were more striking than in the older age groups. The resurgence in cases associated with decreased ITN coverage in 2009 highlights the need for sustained high levels of anti-malarial interventions in Rwanda and other malaria endemic countries.

## Background

Rwanda bordered by Uganda, Burundi, the Democratic Republic of Congo and Tanzania, has an areas of 26,000 sq km and a population of 11 million. Control of malaria, a major public health problem, is integrated into the overall health system through health posts, health centres, district hospitals and referral hospitals complemented by community based treatment using trained health workers. The country is divided into five provinces (North, South, East, West and Kigali) and 30 districts. Rwanda has two distinct malaria epidemiological strata: in two-thirds of the districts, malaria is characterized by seasonal peaks of transmission and in the remaining one-third of districts, malaria transmission is comparatively stable year-round.

Before 2005, preventive interventions were limited to delivery of ITN to pregnant women and children under five through social marketing. In 2001, the country changed its first-line anti-malarial treatment policy from chloroquine to amodiaquine and sulphadoxine-pyrimethamine (AQ+SP), implemented during 2002 – 2005 countrywide; in 2006, the country shifted from AQ + SP to an ACT, artemether-lumefantrine. Rwanda developed its first comprehensive national malaria strategic plan for the period 2005–2010. Subsequently, the National Malaria Control Programme (NCMP), supported by the Global Fund to Fight HIV/AIDS, Tuberculosis and Malaria, the President Malaria Initiative, and other development partners, scaled-up malaria control interventions, with insecticide-treated nets (ITN) and artemisinin-based combination therapy (ACT) as key interventions.

In 2006 and 2007, over three million ITN were distributed through a mass distribution campaign targeting pregnant women and children below five years of age. During 2009–2011, over 6.1 million nets were delivered, with the goal of achieving universal coverage with a ratio of one net per two persons. In August 2007, the country implemented targeted indoor residual spraying (IRS) in selected sectors of the three districts of Kigali and later scaled-up to 32 sectors in 5 districts (Nyagatare, Bugesera, Nyanza, Gisagara and Kirehe). Starting in 2006, the country implemented the policy shift from AQ+SP to an ACT, Artemether-lumefantrine for the treatment of malaria cases in all public health facilities beginning in October 2006. In 2009, the country also implemented nationwide community case management of malaria by training and deploying community health workers (CHW) to test febrile cases using rapid diagnostic tests (RDT) and treat confirmed malaria cases with artemether-lumefantrine. Apart from use by CHWs, RDTs were also used in health centres and higher facilities as adjunct to microscopy in emergency cases, after working hours, when the microscopy was not functional or in situations where case loads were high and exceeded microscopy diagnostic capacity. In hospitals, RDTs may also have been used to reconfirm severe malaria cases when suggestive clinical cases are negative to microscopic diagnosis. However, these RDT results are not reflected in hospital laboratory records, as the counts include microscopy only.

Malaria control has also been an important component of the country's comprehensive poverty reduction strategy, health policy reforms and overall investment in health. Prior to 2004, patients that are 5 years and older were charged for outpatient malaria diagnosis, treatment and admissions, one of several barriers to accessing basic health services for majority of the Rwandese population. To address such problems of access to healthcare, a health insurance scheme (Mutuelle de Santé) was initiated in 1999 and fully scaled up nationwide since 2004. In the Mutuelle de Santé plan, Rwandans pay a USD 15 per year premium and 15% of the medical expenses per healthcare encounter. For people whose inability to pay is certified by local authorities, the government covers all the contributions and expenses.

Rwanda has a functioning disease surveillance system through the Health Management Information System HMIS (SIS). The HMIS includes all public and faith based supported health centres and hospitals, with an average of 95% facilities reporting [1], The system covers a wide range of diseases, including malaria, reported monthly by health district supervisors.

## Methods

### Intervention coverage

Information on malaria control interventions was obtained from NMCP records. The number of ITN distributed in each district during 2000 to 2010 was recorded and the potential proportion of the population protected by ITN by district was calculated for each year, assuming each ITN covered two persons and lasted three years. District level population for each year were derived retrospectively from the 2011 Rwandan census, using the United Nations growth rate for Rwanda [2]. IRS data were not included in the assessment as IRS was limited to only 32 sectors in five districts, covering < 5% of the population and was not consistently applied in all districts or periods during the year. As a measure of ACT availability, the number of ACT monthly stock-outs at health facilities was obtained through the SIS. In addition, potential proportion of malaria cases treated with ACT courses at district level was calculated by dividing total malaria cases reported by total number of ACT courses delivered.

### Malaria morbidity and mortality

A World Health Organization standardized protocol (unpublished) was used for collection of retrospective health facilities based data. The protocol provides sampling methods, case definition of outpatient and inpatient cases and deaths, tallying and recording procedures. Retrospective data were collected from all 40 hospitals in the 30 districts for a period of 2000–2010 using a data abstraction form. Data abstraction teams consisting of two persons visited each hospital and collected data for the relevant indicators from the monthly summary reports covering the study period.

Monthly facility summary reports were used as the main sources of records: i) outpatient records for number of outpatient malaria cases; ii) inpatient discharge records for numbers of malaria inpatient cases and malaria deaths; and iii) laboratory records for the number of positive blood slides and the number of cases that were tested. The slide positivity rate (SPR) was computed by dividing the number of blood slides positive for malaria by the total number of slides examined. Anaemia, though it is a useful indicator [3], was excluded from this analysis, as data were available until 2008 only and results have been reported in a previous study [4].

During 2000–2007, recording of outpatient malaria cases in SIS had differentiated presumed and confirmed cases. However, since 2008 recording of outpatient malaria cases did not differentiate presumed and confirmed cases but classified cases as either uncomplicated or severe malaria. Also, at the hospitals, laboratory records of examined blood slides could not be consistently linked to individual clinical cases nor were the age or admission status of the patient submitting the blood sample recorded. Consequently, assessing trends of confirmed outpatient or confirmed inpatient malaria cases separately throughout the observation period was not possible. In addition, the number of outpatient malaria cases in children under-5 and older ages were less than admission cases in most of the years, indicating incomplete recording. Therefore, the main focus of the study was on total microscopically-confirmed malaria cases, total inpatient malaria cases, and malaria deaths. A confirmed malaria case was defined as a blood slide read as positive for malaria with microscopy at a health facility. An inpatient malaria case was defined as an illness diagnosed at discharge by the treating physician as severe malaria among patients admitted to the inpatient hospital ward. A malaria death was defined as death in an inpatient malaria case attributed to malaria. In the past, health centres in Rwanda had limited inpatient services where inpatient cases and deaths were recorded. However, since 2008, all inpatient services are strictly limited to hospitals only and hence; all retrospective inpatient malaria cases and deaths records collected in this study were from hospitals only. Data from 30 of the total 40 district hospitals in the country that had more than 70% of complete monthly reports per year were included in the analysis. Cases and deaths were stratified by age, less than five years and five or more years. All-cause hospital death and inpatient malaria death rates per 1,000 population were calculated using the district population for each year.

To understand if an increase or decrease in the quality of laboratory testing could have influenced trends in malaria cases, a review was done on the quarterly laboratory quality control system implemented by the National Referral Laboratories (NRL). Started in 2005, the quality control for both health centres and district hospitals was done by the NRL. During 2005–2010, every quarter, 500–700 positive and negative slides from each district hospital and 15 slides from each health centre were collected by the NRL. Collected slides were reread at NRL by expert microscopists and the proportion of results discordant with those of the hospital and health centres were noted.

In order to control for other factors that might influence the observed trends of malaria outpatient cases, admissions and deaths, the following additional comparison indicators were recorded: i) non-malaria outpatient consultations (all-cause outpatient cases minus outpatient malaria cases) to see if changes in malaria cases could be attributable to changes in healthcare seeking; ii) non-malaria admissions (all-cause admissions minus malaria admission) to see if changes in malaria admissions are not due to other factors that also affect trends of all other diseases in a similarly way; iii) non-malaria deaths (all-cause deaths minus malaria deaths) to see if changes in malaria deaths are not due to other factors that also affect trends of all other diseases in a similarly way; and iv) cases, admissions and deaths due to respiratory infections and diarrhoeal diseases. Non-malaria outpatient cases, inpatient cases and deaths were recorded in two age categories, less than five and five years or greater. The number of malaria cases tested and treated at the community level, obtained from Community Health Information System (SISCom), were summed separately and were not included into the outpatient malaria cases at the hospitals or health centres.

The time trends in monthly rainfall in millimetres and land surface temperature in degrees Celsius in 10 sites were obtained from the Rwandan meteorology department to assess the climatic conditions for malaria transmission [5] and control for the potential effect of climatic changes over time in assessing the association between interventions and changes in trends of malaria related indicators.

Results of population based surveys conducted in Rwanda (2000, 2005, 2010 Demographic and Health Surveys [DHS] and interim DHS 2008–9]) [6,7] were reviewed to estimate percentage of households with at least one ITN, parasite prevalence, and all-cause under-five mortality. The total number of deaths in children under five in the country for the time periods 2001–2005 and 2006–2010 were derived from the under-five mortality rate for the corresponding DHS, using the Rwandan crude birth rate to calculate the total number of live births for the five-year period of the survey. The proportion of all under-five deaths in the country which occurred in district hospitals was then calculated for two time periods-2000–2005 and 2006–2010 which correspond approximately to the pre and post intervention period.

### Statistical analysis of time trends

ITN distribution and delivery of artemether-lumefantrine were considered as the two primary malaria control intervention. The pre-intervention period was defined as 2000–2005, before the first mass distribution of ITN and deployment of ACT to the facilities. The main measure on effect of ITN and ACT was evaluated by comparing indicators during 2006–2010 with those in the pre-intervention period 2000—2005 as a simple observed percent change in average of 2006–2010; and using a segmented regression model of an interrupted time series for the corresponding midpoint period (2006–2010) [8]. As additional measures, the same comparisons were also done for each post-intervention year to allow assessment of the changes at a given post-intervention period. This model corrects for autocorrelation [9,10] and adjusts the change estimate allowing for (1) possible time trend of the indicator during the pre-intervention period; (2) a possible immediate drop or rise of the indicator following the start of the intervention, and (3) an effect of the intervention on the post intervention time trend of the indicator.

The effect of malaria interventions using the model was calculated as the percent difference between observed and the predicted indicator levels for the midpoint during 2006–2010, assuming a continuation of the pre-intervention time trend throughout 2010 if there had been no intervention. The 95% confidence intervals (CI) around effect estimates were computed using the CI around the regression coefficient. A positive percent difference indicates an increase in the indicator and a negative percent difference a decrease in the indicator. A percentage difference with a CI that does not include zero in the range was considered as being a significant change in the indicator.

## Results

### Interventions

The proportion of the population potentially protected by ITNs in the districts increased from nearly zero in early 2005 to 38% in 2006 and 59% in 2007 following the first mass distribution campaign to children under-5 and pregnant women (Additional file 1: Table S1). Coverage fell in 2009 to 32% as ITN exceeded their assumed durability, and then increased in 2010 to 76% following the second mass distribution campaign targeting all ages (Additional file 1: Table S1). The type of the nets used in mass and routine distribution during 2006–2010 was Permanet® except 2009 where Olyset® was used for mass distribution. In household surveys, the percentage of households who owned at least one ITN increased from 15% in the Rwanda DHS 2005 to 57% in 2007–8 DHS and to 82% in Rwanda DHS 2010; and the percentage of children under-5 who slept under an ITN the previous night increased from 13% in 2005 to 58% in both 2007–8 and 2010 [11,12]. The supply of artemether-lumefantrine at health facilities was assumed to be constant throughout the post intervention period as there were no large or repeated stock-outs at facilities recorded after its introduction in 2006 and distributed ACT courses as a percentage of malaria cases remained high. The percentage of health facilities reporting no stock out of ACT for more than a week during the past three months in 2009, 2010 and 2011 were 89%, 95% and 99% respectively. The potential proportion of malaria cases treated with ACT at district level for 2008, 2009 and 2010 was 105%, 97% and 93% respectively (Additional file 1: Table S1). Districts in the Eastern province had relatively lower number of ACT courses compared to the number of malaria cases reported.

### Trends on the malaria outpatient cases, inpatient cases and deaths and non-malaria indicators during 2000–2010

**Figure 1:**
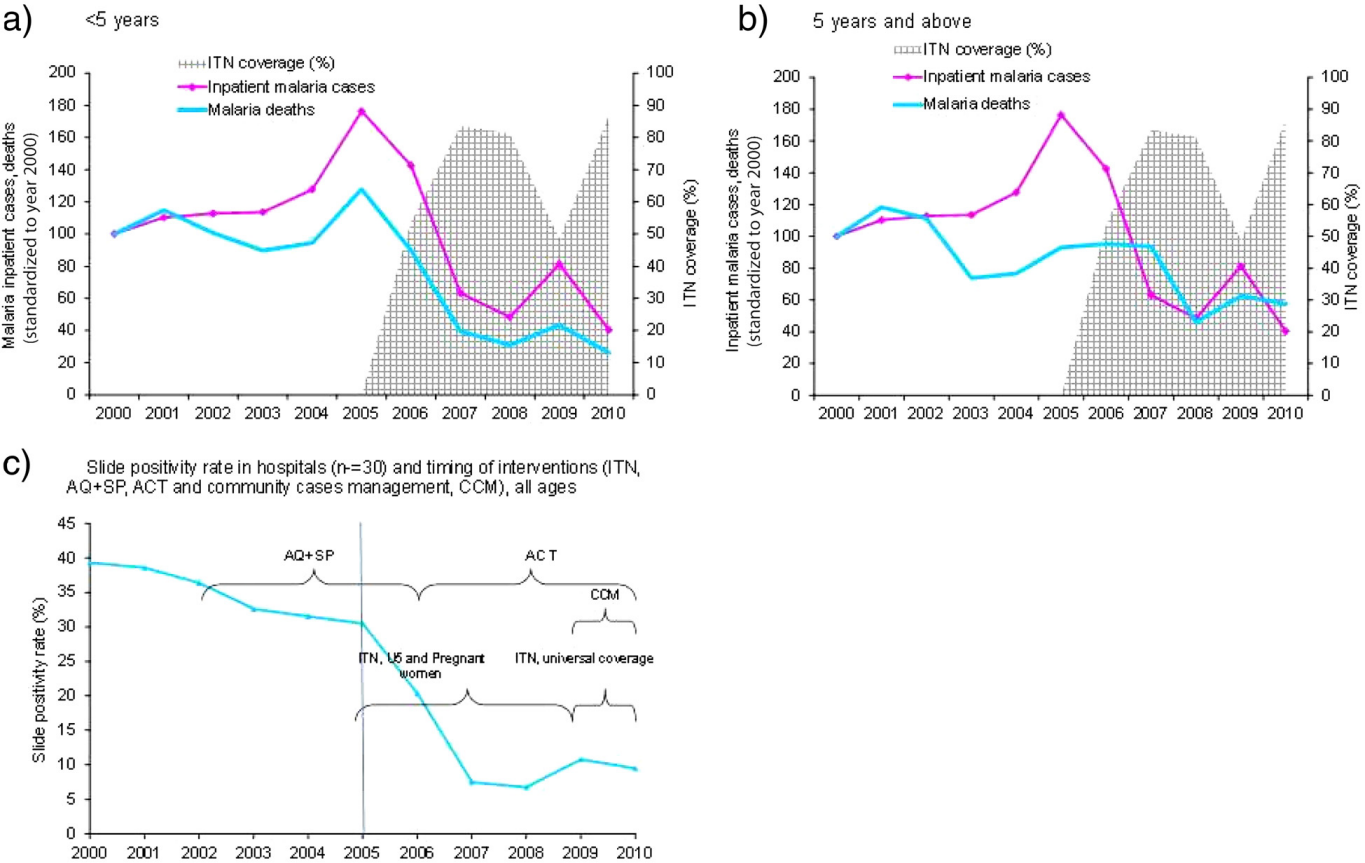
Trends of malaria cases and deaths by age group, slide positivity rate in district hospitals and timing of interventions, 2000–2010, Rwanda.

**Figure 2:**
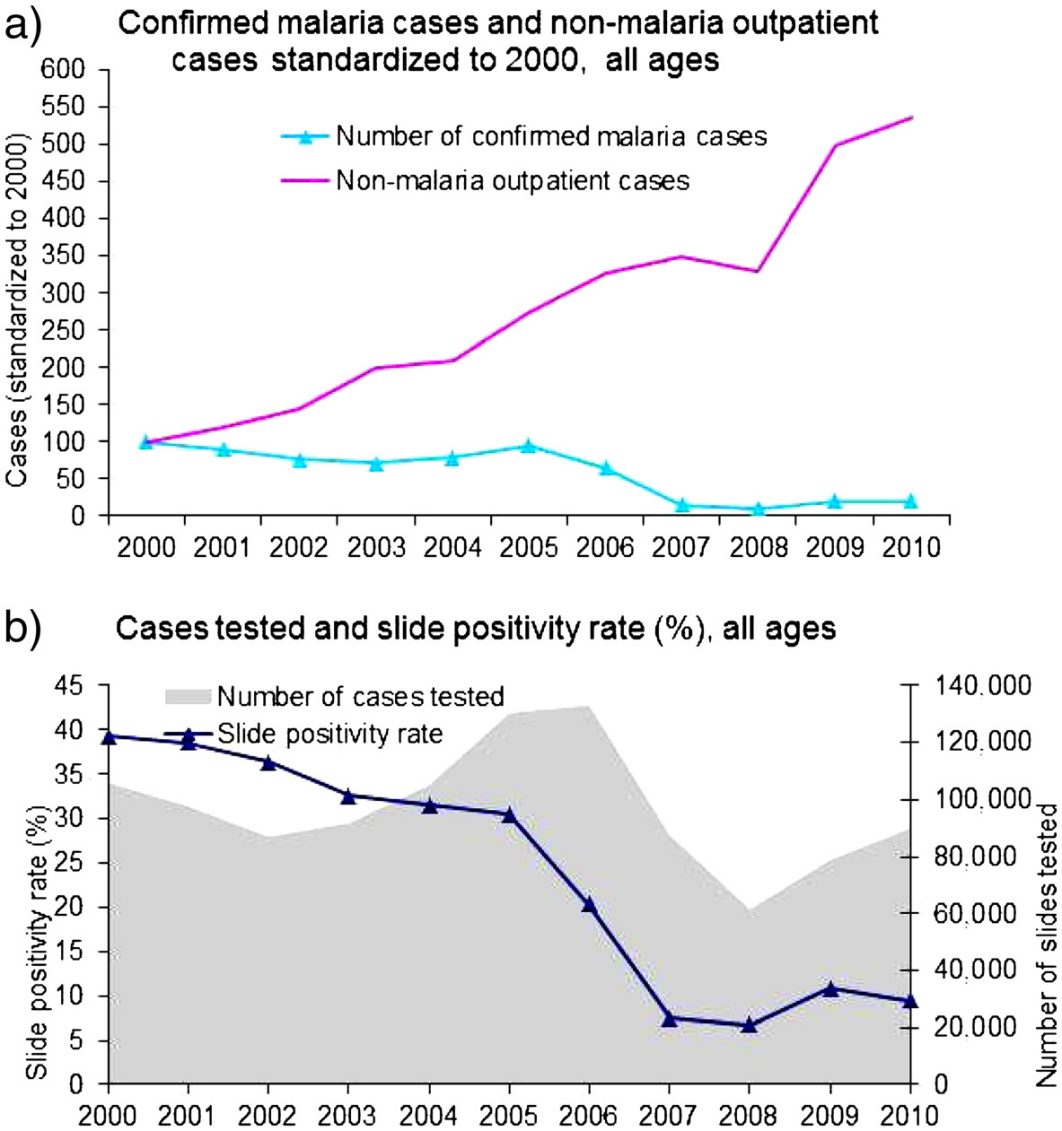
Confirmed malaria cases, non-malaria outpatient cases, number of cases tested and slide positivity rate in all ages, 30 hospitals, 2000–2010, Rwanda.

**Figure 3:**
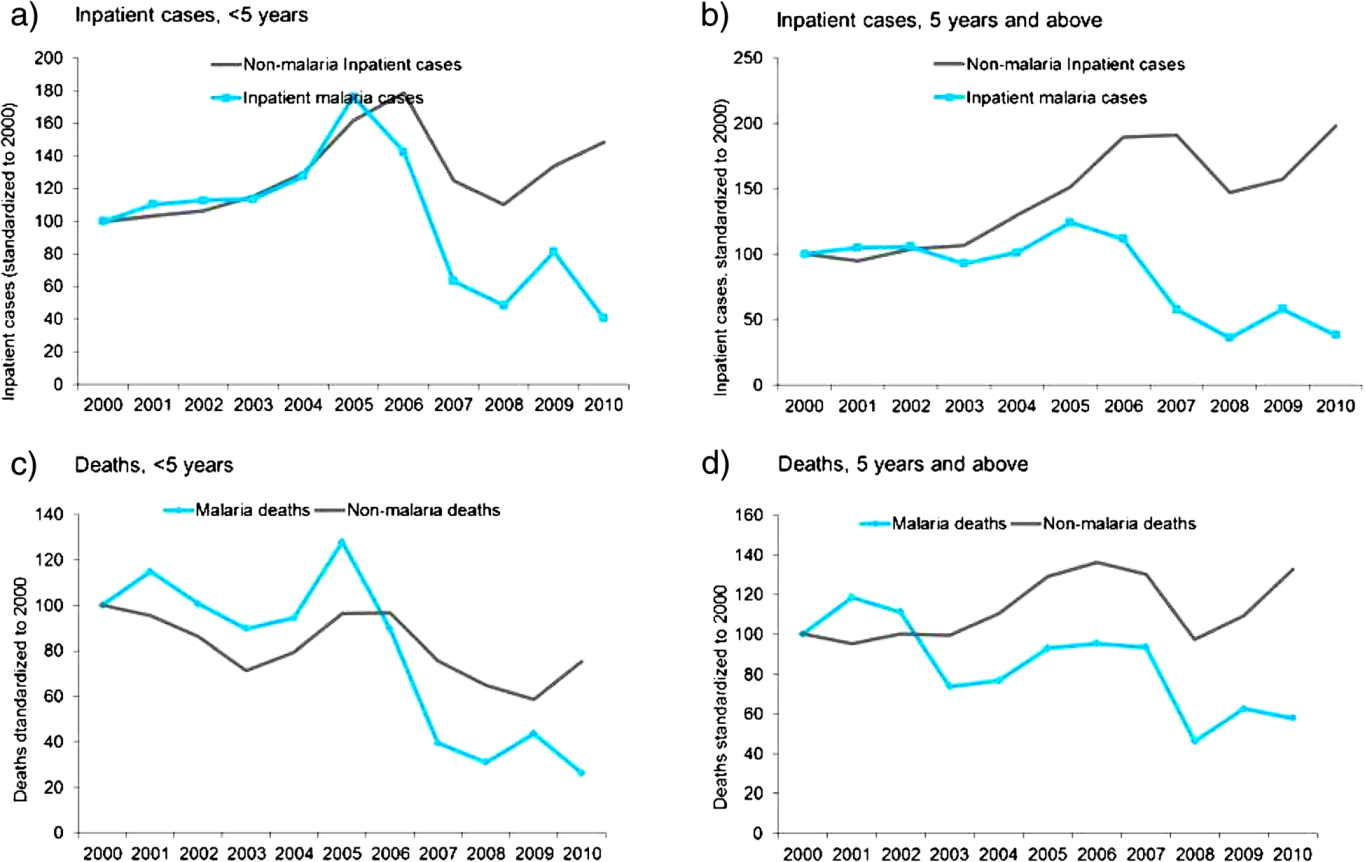
Inpatient malaria and non-malaria cases and deaths in 30 hospitals by age, 2000–2010, Rwanda.

Temporally, malaria related indicators (slide positivity rate, inpatient cases and deaths) followed similar trends of decline during 2000–2010 in children under five and 5 years and above (Figure 1). In the pre-intervention period, inpatient cases and malaria deaths decreased slightly in 2002–2004 and increased during 2005–2006. Inpatient malaria cases for all ages decreased substantially in 2006 and 2007 as ITN coverage increased, and then increased slightly in 2009 as operational coverage of ITN decreased. Cases decreased in 2010 as operational ITN coverage increased following the second mass ITN distribution. In general, malaria indicators for outpatients (Figure 2) and inpatients (Figure 3) decreased over time while non-malaria indicators did not. The CCM programme led to diagnosis and treatment of approximately 48,000 confirmed cases in 2009 and 53,000 in 2010 in the community, though this occurred two years after the number of confirmed malaria cases at the hospital decreased in 2007 (Table S2).

**Figure 4:**
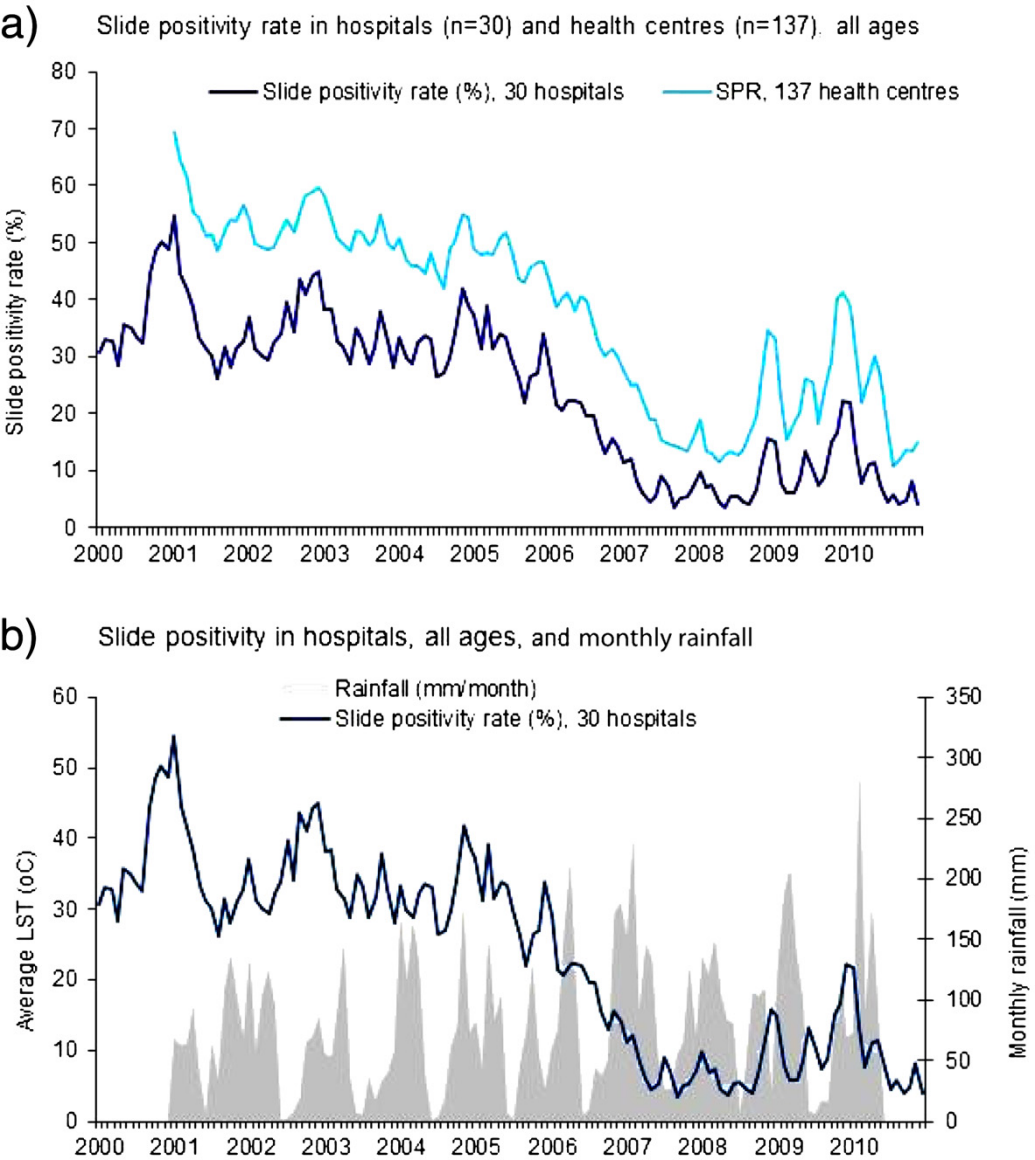
Trends on monthly slide positivity rate in hospitals, health centers, and rainfall, 2000–2010, Rwanda.

The number of patients tested for malaria for all ages in the visited hospitals varied during the observed period, while slide positivity rates fell (Figure 2b). The number of cases tested in the hospitals decreased from 123,207 in 2006 to 58,239 in 2008 (53% decline) which corresponded with a fall in SPR from 31% to 7% during the same period. Slide positivity rate in 137 health centres with complete monthly microscopic data obtained from the SIS revealed a similar decrease in slide positivity rate from an average of 53% during the baseline period to 19% in 2010 (Figure 4a). During the 2005–2011, the laboratory quality control system for district hospitals showed discordant rates ranging from 1.4% - 4.3%. Since the laboratory quality control started in 2005, data on earlier years was not available.

### Comparing pre and post intervention periods, by age group

#### All ages

The number of confirmed malaria cases declined from an average of 35, 688 during 2000– 2005 to 8,517 in 2010, representing an observed 76% decline from the 2000–2005 average and a 73% decline from the predicted value for 2010 in the model (CI, 12%-91%) (Additional file 2: Table S2). The SPR in all ages fell 58% (CI, 47%-66%). Malaria inpatient cases in all ages decreased 76% (CI, 49% – 88%). During the same period malaria deaths decreased 47% (CI, 47% - 81%). The number of outpatient malaria cases was less than inpatient cases, particularly for children under-5 years in hospitals, as most of the uncomplicated malaria cases were treated at small dispensary attached to each hospital providing outpatient services.

#### Children under five

Malaria outpatient cases (both confirmed and presumed cases), declined 90% (CI, 80%-95%), inpatient malaria inpatient cases decreased 82% (CI, 61-92%) and malaria deaths declined 77% (CI, 40% – 91%) during the pre- and post-intervention period.

#### Five years and above

Malaria outpatient malaria cases declined 70% (CI, 30%-93%), inpatient malaria cases decreased 69% (CI, 42-83%) and malaria deaths declined 36% (CI, 7% -67%) during the pre- and post-intervention period.

### Non-malaria indicators

Non-malaria outpatient cases, inpatient cases and deaths in both children under-5 and older ages generally followed similar trend during pre-intervention period and declined briefly in 2007 and 2008 but increased again in 2009 and 2010. Although statistically insignificant, non-malaria deaths in children under-5 years of age showed declining trend together with the malaria deaths (Figure 2, Figure 3 and Additional file 2: Table S2).

### Effect of rainfall anomalies by year

There were no noticeable anomalies in rainfall and temperature that could plausibly affect the normal seasonal trends of slide positivity rate and inpatient malaria cases (Figure 4b). The highest rainfall recorded was in 2010. There was normal seasonality of rainfall between 2007– 2008, the period during which both malaria slide positivity rate and inpatient cases were at their lowest. Mean Land Surface Temperature at night time during pre and post intervention were similar, 13.45°C (12.14-14.79) °C and 13.49°C (12.38-15.07).

### Spatial difference in intervention effect

**Figure 5:**
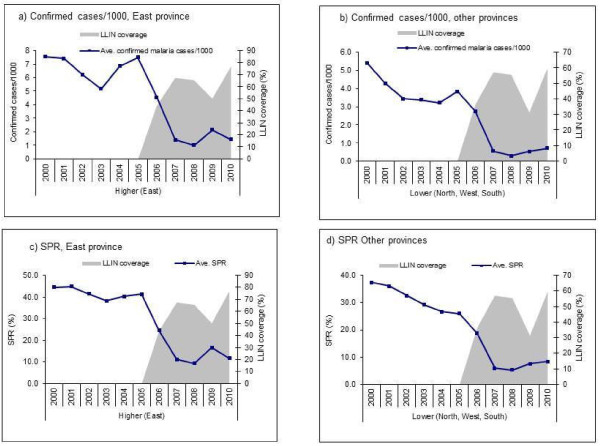
Trends in average confirmed malaria case/1,000 and slide positivity rate in hospitals and LLIN coverage, by East and other provinces, 2000–2010 Rwanda.

**Figure 6:**
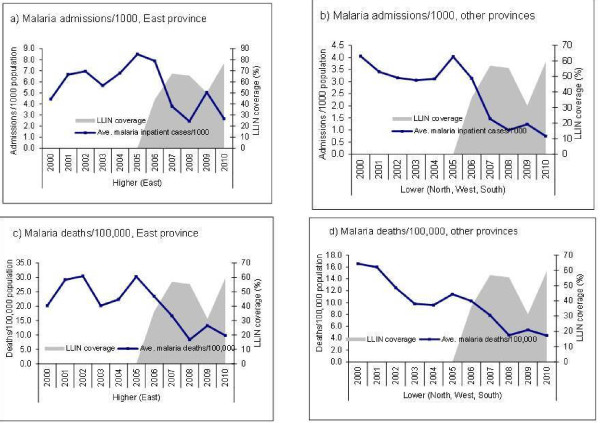
Trends on average malaria admissions/1,000, malaria deaths/100,000 in hospitals and LLIN coverage, by East and other provinces, 2000–2010, Rwanda.

**Figure 7:**
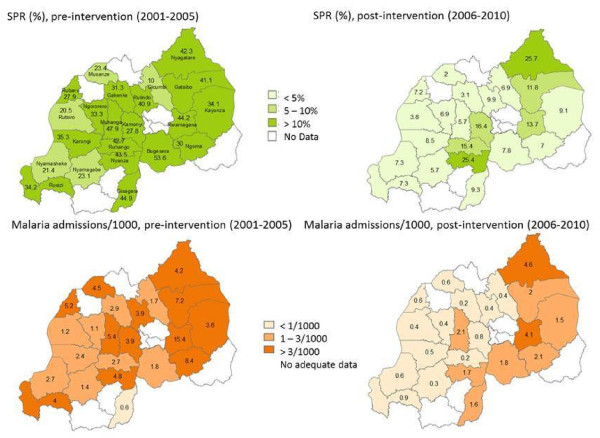
Slide positivity rate and malaria admission rate by district during pre- and post-intervention period, 2000-2010, Rwanda.

Hospitals in the East province had higher rates of malaria admissions (6/1,000 population) and deaths (25/100,000) in the pre-intervention period than hospitals in the other provinces (West, North and South), which had admission and death rates of 3/1,000 and 11/100,000 respectively. SPR in the East was 41% while the other provinces had 36% in the pre-intervention period. When provinces were grouped into regions with higher (East) and lower (North, South, West) malaria burden based on baseline malaria admissions, death and slide positivity rates, similar declines in confirmed malaria cases, SPR, malaria hospital admissions and deaths were temporally associated with increased ITN coverage in each region (Additional file 1: Table S1, Figure 5, and Figure 6). The slide positivity rate and incidence of malaria admissions per 1000 at district level during pre- and post-intervention periods are presented in map (Figure 7). Malaria incidence in some districts in the East and South provinces remained relatively high during the post intervention period.

In further analysis, in the pre-intervention period, the increase in inpatient malaria cases and deaths during 2003–2005 was dominated by three hospital: Rwamagana, Ngarama and Kibungo (37% of all malaria admissions and 44% of malaria deaths), all in the East province. All other hospitals also had a slight increase in malaria admissions and deaths during this period. In the post-intervention period the increase in 2009 was dominated by three hospital Rwamagana, Ngarama and Gakoma (51% of admissions and 34% of malaria deaths), again all in the East province (Figure 7).

### Parasite prevalence

Plasmodium parasite prevalence was available from two household surveys during the observation period, both conducted during the post intervention period. Parasite prevalence in children under five-years of age was 2.6% in the 2007 [11] and 1.4% in the 2010 [12].

### Population mortality

**Figure 8:**
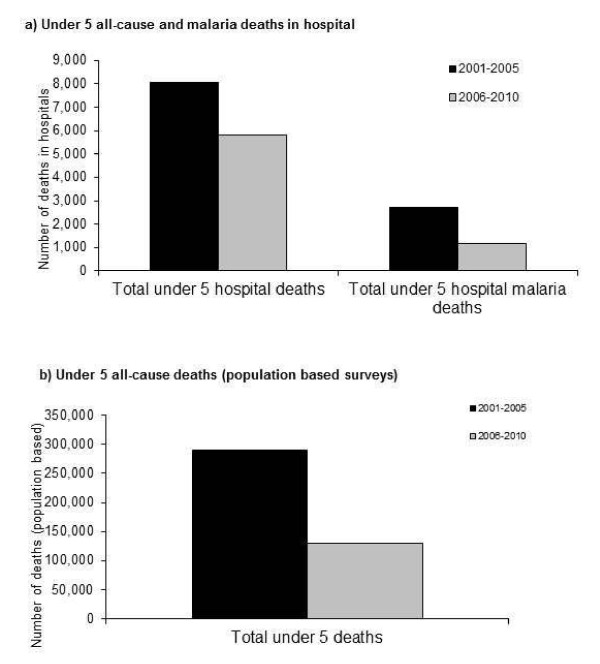
Trends in under-five child mortality (in population surveys, DHS2000, 2005, 2010), all-cause under-five child mortality and malaria deaths in hospitals (n = 30), 2000–2010, Rwanda.

All-cause mortality for children under-five in Rwanda measured in the DHS declined from 152 per 1,000 live births during 2001–2005, to 76 per 1,000 live births during 2006–2010. The estimated total number of deaths in children under five years was 289, 039 during 2001–2005 and 129,936 during 2006–2010, representing a decline of 55%. Comparing hospital deaths to population deaths for the two periods, all-cause deaths in the 30 hospitals in this study represent 2.8% of all deaths in children under-five in the country for the years 2001–2005, and 4.5% during 2006–2010. Total hospital deaths and malaria hospital deaths both declined from the pre-intervention to the post intervention periods (Figure 8): five-year cumulative all-cause hospital deaths declined 28% (from 8,051 to 5,801) and hospital malaria deaths declined 56% (2,724 to 1,187). Approximately 68% of the decline in the number of all-cause hospital deaths was due to the decline in the number of malaria deaths.

## Discussion

This assessment of the impact of malaria control interventions using hospital data shows a decline in the confirmed malaria cases, admissions and deaths following scale-up of ITN coverage and availability of ACT as first-line malaria treatment. Five years after intervention scale-up began, total confirmed cases, slide positivity rates, malaria admissions, and malaria deaths, fell by >50% across all ages. The slide positivity rate and number of malaria inpatients decreased significantly every year post-intervention although the decline in 2009 was less. The declines in malaria indicators in children under-5 years during 2007–2010 were more striking than in the older age group. Malaria admissions and deaths in children under-five declined by >70% five years post-intervention while malaria deaths in 5 years and above declined approximately 30%. The larger decline in deaths among children under-may be due, in part, to the fact that this was initially the main target group for ITN distribution. From the data presented, it is not clear whether older ages have become more vulnerable than before due to the gradual loss of partial immunity as malaria burden decreased following intensified malaria control. The decline in malaria indicators occurred in the face of increased healthcare utilization, and across all age groups and regions with different levels of baseline malaria burden. No changes in rainfall or temperature were identified that could explain the observed differences in malaria indicators. Although unmeasured factors may have contributed to the decline in malaria in health facilities in Rwanda, available health facility data indicate that this decline coincided with the scale-up of malaria interventions mainly with ITN distribution. A similar decline in slide positivity rates in outpatient health centres suggests that the decline in malaria was not limited to district hospitals. In this study, changes were measured by case counts, as the precise catchment population for each hospital was not known. Taking into account the natural increase in population in Rwanda during the study period and the increase in health facility utilization, the decline in malaria indicators measured as rates may have been steeper than shown here.

Declines in hospital deaths attributed to malaria contributed substantially to the decline in all-cause hospital deaths in children in the 30 hospitals in the study. Total all-cause hospital deaths in children under-five (derived from household surveys) fell from 8,051 in 2001–2005 to 5,801 in 2006–2010 (28% decline) during the study period and malaria accounted for 68% of that decline. Although all-cause hospital deaths in the 30 hospitals in this study represented a small fraction of the total estimated deaths in the population for Rwanda, both all-cause mortality in children under-five measured in hospitals and at the population level measured through household surveys declined during the study period. The estimated decline in all-cause under-5 mortality was greater in the population (56%) than observed in the district hospitals (28% in 2010). The magnitude of the decline in all-cause under-5 mortality and its associations with reductions in malaria mortality observed in Rwanda is consistent with what would be expected, given the proportion of under 5-mortality associated with malaria previously observed for sub-Saharan African countries with stable malaria [13].

Despite the apparent overall reduction in malaria over the observed period, smaller year-to-year changes highlight the variability in malaria incidence over time, and the potential for surges in malaria transmission if coverage of preventive interventions decreases or anti-malarial treatments become ineffective [14]. The brief decrease and increase in malaria indicators during 2002–2005 appears to correspond temporally to the change in recommended first line anti-malarial treatment in Rwanda, from chloroquine to AQ-SP, and may relate to the brief effectiveness of AQ-SP and rapid decline in its efficacy during the same period [15,16]. The increase in malaria cases in 2009 may have resulted from reduced effective coverage with ITN due, in part, to delays in replacing ITNs originally delivered in 2006. By 2009, these ITNs were likely to have been rendered less effective due to reduced physical integrity or insecticide efficacy after their estimated three-year effective lifespan. Replenishment of ITN through a second campaign in 2009 and 2010 and resulting increased effective ITN coverage coincided with stabilization of malaria cases to levels similar to 2007 and 2008. The overall increase in ITN coverage is also confirmed in household surveys [11,12].

Assessments of the impact of malaria control activities using facility data have been conducted in other high-endemic sub-Saharan African countries including Zanzibar [17], The Gambia [18] and in Sao Tome and Principe [19]. Bioko Island in Equatorial Guinea and Zambia used household surveys to assess the effect of the interventions [20]. In Zambia, parasitaemia in children under-5 decreased from 22% nationwide in 2006 to 10% in 2008 following scale-up of malaria interventions [21]. In a follow up survey in 2010, parasitaemia increased to 16%, which was attributed in part to lack of timely replacement of ITN [22].

When interpreting the results presented, the limitations of any health facility based data in assessing trends in disease occurrence should be kept in mind, particularly changes in service utilization and reporting completeness, [23]. In this study, an attempt has been made to address these problems by focusing on health facilities with complete reporting as well as checking trends in diagnostic effort. In the present study, the declines in malaria cases in the 30 hospitals were observed against increases in non-malaria attendances, non-malaria admissions and non-malaria deaths. In children under-5 years though insignificant, non-malaria deaths declined 9% (observed) in the mid period 2006–2010. Given the limitations of missing the data, it was difficult to assess whether 10 districts hospitals excluded from the analysis had different trends in malaria cases and details. The effect of community case management of malaria on trends in district hospital malaria cases appears to have been small. The decline in malaria cases and deaths, starting 2007, occurred two years before the scale up of community case management, which started in 2009. Also, given health seeking behaviour in Rwanda, in lieu of community treatment, it is likely that approximately half of those patients may not have sought any treatment, and those who did seek treatment would have been seen at various levels of the health system, with only a small fraction (1%) presenting for care at the hospitals [12].

Information on testing practices at the district hospitals was limited. Although the number of tests performed was recorded, the number of suspected cases presenting to health facilities that should have been tested for malaria was not recorded in standard reporting forms and therefore, we could not assess whether the proportion of suspect patients tested changed over time. The annual numbers of slides examined varied somewhat throughout the pre- and post-intervention periods; however, it appears that malaria diagnostic testing did not dramatically change during the observed period. The declines in the number of patients tested for malaria from 2006 to 2008 was consistent with the decline in the number of suspect malaria cases represented by the decline in the slide positivity rate. The omission of the recording and reporting of suspect malaria cases demonstrates that deficiencies may be present in otherwise well functioning surveillance systems for malaria. Tracking suspect malaria patients, consistent recording of confirmed malaria cases and linking laboratory results to individual cases would improve the routine information system for tracking malaria in Rwanda.

While these results may represent true time trends in malaria at the health facilities, they may not reflects trends of malaria burden at the population level. The effect of ITN, which are widely distributed in the community may be more consistent when measured at health facilities than the effect of ACT, whose effect is dependent on healthy facility access which may vary in the community. Although interrupted time-series analysis controls for baseline trends when estimating effects of interventions and is preferable to methods such as comparison of changes in proportions before and after intervention, estimates of overall effects do involve extrapolation, are subject to some degree of uncertainty, and hence, results should be cautiously interpreted.

## Conclusions

A greater than 50% decline in malaria cases and deaths was observed following scale-up of mainly ITN and ACT in Rwanda since 2005. The decline occurred among both children under-5 and in those 5 years and above, while hospital utilization increased and suitable conditions for malaria transmission persisted. Declines in malaria indicators in children under-five were more striking than in the older age groups. The resurgence in cases associated with decreased ITN coverage highlights the need for sustained high levels of anti-malarial interventions in Rwanda and other malaria endemic countries.

It appears that data collected for routine surveillance can be used to assess the effect of malaria interventions. When using this approach, the limitations of routine facility data must be understood and accounted for. Control of malaria in Rwanda is dependent on sustained coverage of effective interventions and strengthened surveillance.

## Competing interests

The authors declare that they have no competing interests.

## Authors’ contributions

MA and CK carried out the study design, data collection, analysis and drafting and overall coordination of writing up-of the paper. AR, AK, MM helped in data collection and editing; and field supervision. SF and KG helped in reviewing the paper and programme interventions in Rwanda. RW helped in data management and analysis. ML and RC helped in reviewing the manuscript. RN provided overall technical guide and reviewed the paper. NF, JN, DN and IU participated in the data collection, analysis and review of the paper. AB provided guidance on the objectives of the study, study design, interpretation of results and reviewed of the paper. All authors read and approved the final manuscript.

## Supplementary Material

Additional file 1 **Table S1.** Percentage change in laboratory confirmed malaria cases, inpatient cases and deaths in 2010 compared to pre-intervention period (2000–2005) in hospitals and potential proprtion of population protected by ITN, Rwanda, 2000-2010. Click here for file

Additional file 2 **Table S2.** Percentage change in malaria and non-malaria indicators in post intervention years (2006-2010) compared to pre-intervention period (2000–2005), for all ages and <5 years, in 30 of the 40 hospitals, Rwanda, 2000-2010. Click here for file
