# Supplementary material for: Trends in malaria cases, hospital admissions and deaths following scale-up of anti-malarial interventions, 2000–2010, Rwanda
Source: Malar J. 2012 Jul 23;11:236. doi: 10.1186/1475-2875-11-236 (PMC3502144; doi:10.1186/1475-2875-11-236)
Supplement: Additional file 1 — Table S1. Percentage change in laboratory confirmed malaria cases, inpatient cases and deaths in 2010 compared to pre-intervention period (2000–2005) in hospitals and potential proprtion of population protected by ITN, Rwanda, 2000-2010. [file 1475-2875-11-236-S1.pdf]

Table 1. Percentage change in laboratory confirmed malaria cases, inpatient cases and deaths in 2010 compared to pre-intervention period (2000–2005) in hospitals and potential proprtion of population protected by ITN, Rwanda, 2000-2010

				Microscopy: Confirmed (Positive)			Microscopy: Slide positivity rate (SPR)			Inpatient malaria cases			Malaria deaths			Confirmed cases/1000 population			Inpatient malaria cases/1000 population			Malaria deaths/100,000 population			Potential proportion of population protected by ITN (%)					Potential proportion of malaria cases treated with ACT at district level (%)		
Emdemicity	Province	District	Hospital	Average (2000-05)	2010	% chang e	Avera ge (2000-05)	2010	% change	Average (2000-05)	2010	% change	Avera ge (2000-05)	2010	% change	Average (2000-05)	2010	% change	Avera ge (2000-05)	2010	% chan ge	Averag e (2000-05)	2010	% change	2006	2007	2008	2009	2010	2008	2009	2010
High	East	Bugesera	Nyamata	2,179	334	-85	53	8	-85	592	631	7	23	34	48	8	1	-88	2	2	-14	8	10	21	46	69	67	33	27	93	91	76
High	East	Gatsibo	Kiziguro	955	336	-65	45	12	-74	1,367	946	-31	35	31	-11	3	1	-71	4	3	-43	12	8	-28	43	65	63	61	57	86	123	64
High	East	Gatsibo	Ngarama	1,313	435	-67	36	12	-67	2,746	562	-80	132	48	-64	4	1	-73	9	2	-83	43	13	-70	43	65	63	61	57	86	123	64
High	East	Kayonza	Gahini	777	476	-39	36	9	-75	776	413	-47	33	20	-40	3	2	-49	3	2	-56	15	7	-52	45	71	69	36	95	93	70	65
High	East	Ngoma	Kibungo	1,998	274	-86	30	7	-76	1,916	638	-67	79	35	-56	8	1	-89	7	2	-72	31	11	-64	44	68	66	36	91	137	110	84
High	East	Nyagatare	Nyagatare	2,107	527	-75	46	26	-44	1,156	1,536	33	68	42	-39	8	2	-80	4	5	9	25	13	-50	42	67	65	112	106	80	92	89
High	East	Rwamagana	Rwamagana	2,971	807	-73	43	14	-68	3,352	1,178	-65	93	8	-91	13	3	-78	14	4	-71	39	3	-93	46	67	65	30	90	104	91	86
**Subtotal (high transmission, East province)**				**12,302**	**3,189**	**-74**	**41**	**12**	**-70**	**11,904**	**5,904**	**-50**	**464**	**218**	**-53**	**7**	**1**	**-79**	**6**	**3**	**-59**	**25**	**9**	**-62**	**44**	**67**	**65**	**53**	**75**	**103**	**91**	**81**
Low	North	Gakenke	Ruli	769	34	-96	35	3	-92	1,466	61	-96	40	4	-90	2	0	-96	4	0	-97	12	1	-92	38	59	58	32	76	74	112	100
Low	North	Gakenke	Nemba	2,166	125	-94	27	4	-87	548	84	-85	38	8	-79	6	0	-95	2	0	-88	11	2	-83	38	59	58	32	76	74	112	100
Low	North	Gicumbi	Byumba	186	158	-15	11	7	-37	668	175	-74	41	18	-56	0	0	-32	2	0	-79	11	4	-65	33	53	52	29	22	62	76	121
Low	North	Musanze	Ruhengeri	1,450	123	-92	24	2	-92	1,470	240	-84	44	12	-73	4	0	-93	4	1	-87	13	3	-77	38	61	59	32	24	101	113	84
Low	North	Rulindo	Rutongo	1,113	117	-89	41	10	-76	1,006	117	-88	23	10	-57	4	0	-91	4	0	-90	9	3	-65	28	48	47	20	40	161	153	113
Subtotal, North province				***5,684***	***557***	***-90***	***138***	***25***	***-82***	***5,159***	***677***	***-87***	***185***	***52***	***-72***	***4***	***0***	***-92***	***3***	***0***	***-89***	***11***	***3***	***-77***	***35***	***56***	***55***	***29***	***48***	***108***	***114***	***106***
Low	South	Gisagara	Gakoma	1,246	234	-81	46	9	-80	183	554	203	9	9	5	4	1	-85	1	2	147	3	3	-13	37	58	56	36	33	127	125	101
Low	South	Kamonyi	Remera-Rukoma	1,782	442	-75	28	16	-41	1,077	284	-74	50	10	-80	6	1	-80	4	1	-78	18	3	-84	48	68	67	29	79	97	91	97
Low	South	Muhanga	Kabgayi	2,208	305	-86	48	6	-88	1,804	790	-56	40	34	-14	7	1	-89	6	2	-64	13	9	-28	52	70	68	27	76	125	90	98
Low	South	Nymagabe	Kaduha	273	135	-51	25	5	-79	369	53	-86	15	3	-80	1	0	-59	1	0	-88	5	1	-83	41	64	62	38	83	83	87	93
Low	South	Nymagabe	Kigeme	585	69	-88	22	6	-72	474	147	-69	35	20	-43	2	0	-90	2	0	-74	11	5	-52	41	64	62	38	83	83	87	93
Low	South	Nyanza	Nyanza	1,281	788	-38	45	25	-44	1,255	500	-60	41	30	-27	5	3	-49	5	2	-67	17	10	-41	37	55	53	30	88	93	92	98
Low	South	Ruhango	Gitwe	1,390	310	-78	43	15	-64	738	73	-90	22	-	-100	5	1	-82	3	0	-92	8	0	-100	51	71	69	33	95	92	139	539
Subtotal, South province				***6,129***	***1,739***	***-72***	***168***	***59***	***-65***	***4,978***	***1,774***	***-64***	***180***	***97***	***-46***	***4***	***1***	***-76***	***4***	***1***	***-71***	***13***	***6***	***-56***	***44***	***64***	***62***	***32***	***82***	***101***	***95***	***97***
Low	West	Karongi	Kibuye	645	178	-72	28	6	-80	634	183	-71	22	15	-32	2	0	-78	2	1	-77	8	4	-46	17	39	38	34	84	86	91	94
Low	West	Karongi	Kirinda	1,136	114	-90	31	7	-76	1,074	141	-87	20	5	-75	4	0	-92	4	0	-89	7	1	-80	17	39	38	34	84	86	91	94
Low	West	Karongi	Mugonero	360	214	-41	49	12	-75	483	186	-62	18	5	-72	1	1	-51	2	1	-68	6	1	-77	17	39	38	34	84	86	91	94
Low	West	Ngororero	Muhororo	1,016	154	-85	52	6	-89	432	199	-54	17	10	-42	3	0	-88	1	1	-63	6	3	-55	35	56	55	32	90	79	98	80
Low	West	Nyamasheke	Bushenge	279	89	-68	24	6	-74	751	207	-72	45	19	-58	1	0	-75	2	0	-78	13	4	-66	30	51	50	32	28	114	114	84
Low	West	Nyamasheke	Kibogora	1,447	596	-59	25	8	-67	1,320	290	-78	48	8	-83	4	1	-67	4	1	-82	14	2	-87	30	51	50	32	28	114	114	84
Low	West	Ngororero	Kabaya	259	151	-42	20	8	-58	290	75	-74	17	14	-15	1	0	-53	1	0	-79	6	4	-33	35	56	55	32	90	79	98	80
Low	West	Rubavu	Gisenyi	843	403	-52	30	7	-76	1,688	227	-87	91	24	-74	3	1	-62	5	1	-89	30	6	-79	33	57	55	34	95	80	91	93
Low	West	Rusizi	Gihundwe	1,347	133	-90	25	5	-80	1,773	213	-88	35	19	-45	4	0	-92	5	0	-90	10	4	-55	32	54	53	32	28	56	74	100
Low	West	Rusizi	Mibirizi	1,060	263	-75	42	10	-77	1,064	576	-46	34	41	20	3	1	-79	3	1	-56	10	9	-2	32	54	53	32	28	56	74	100
Low	West	Rutsiro	Murunda	695	194	-72	23	4	-83	421	132	-69	14	10	-29	3	1	-78	2	0	-75	5	3	-44	24	45	44	32	28	145	122	83
Subtotal, West province				***4,267***	***939***	***-78***	***197***	***39***	***-80***	***3,475***	***1,234***	***-64***	***104***	***71***	***-32***	***3***	***1***	***-78***	***3***	***1***	***-80***	***10***	***4***	***-62***	***27***	***49***	***48***	***33***	***61***	***113***	***102***	***110***
**Subtotal (low, transmisison North, South and West provonice together)**				**6,358**	**1,713**	-73	**36**	**8**	-79	**5,428**	**1,707**	-69	**174**	**94**	-46	**4**	**1**	-80	**3**	**1**	-78	**11**	**4**	-65	**34**	**55**	**54**	**32**	**63**	**107**	**104**	**104**
**Total**				**28,381**	**6,423**	-77	**35**	**10**	-73	**25,516**	**9,589**	-62	**932**	**438**	-53	**4**	**1**	-80	**4**	**1**	-71	**14**	**5**	-64	**38**	**59**	**58**	**32**	**76**	**105**	**97**	**93**

A negative percentage indicate a decrease of the indicator.
