# Supplementary material for: Trends in malaria cases, hospital admissions and deaths following scale-up of anti-malarial interventions, 2000–2010, Rwanda
Source: Malar J. 2012 Jul 23;11:236. doi: 10.1186/1475-2875-11-236 (PMC3502144; doi:10.1186/1475-2875-11-236)
Supplement: Additional file 2 — Table S2. Percentage change in malaria and non-malaria indicators in post intervention years (2006-2010) compared to pre-intervention period (2000–2005), for all ages and <5 years, in 30 of the 40 hospitals, Rwanda, 2000-2010. [file 1475-2875-11-236-S2.pdf]

Table 1. Percentage change in malaria and non-malaria indicators in post intervention years (2006-2010) compared to pre-intervention period (2000–2005), for all ages and <5 years, in 30 of the 40 hospitals, Rwanda, 2000-2010.

	Indicator	Pre-intervention average (2000-05)	Post intervention average (2006-10)	Values observed in 2010	Observed change in pre-intervnetion (2000-2005) vs 2010 (%)	Observed change in pre-intervnetion (2000-2005) vs average of post-intervention (2006-2010) (%)	Changes in predicted versus observed values in post-intervention years (correcting for autocorrelation) Post-intervention year: Year 1 (2006)	Year 2 (2007)	Year 3 (2008)	4 years (2009)	Year 5 (2010)	Midpoint change during 2006-2010[a]
**CHILDREN UNDER 5 YEARS**												
**All-cause outpatient**	All-cause outpatient cases	21,002	40,777	48,991	133	48	11% (-31% – 77%)	-4% (-43% – 59%)	-30% (-60% – 22%)	-6% (-48% – 72%)	-26% (-61% – 41%)	29% (-19% – 107%)
	All-cause admissions	32,847	30,226	27,536	-16	-9	9% (-32% – 74%)	-40% (-65% – 1%)	-54% (-74% – -18%)†	-44% (-70% – 5%)	**-53% (-76% – -8%)†**	-26% (-54% – 19%)
	All-cause deaths	1,635	1,160	1,073	-34	-41	6% (-46% – 107%)	-26% (-65% – 55%)	-37% (-72% – 43%)	-37% (-74% – 54%)	-28% (-73% – 89%)	-26% (-62% – 44%)
**Malaria**	Outpatient malaria cases	3,721	6,644	2,293	-38	44	-4% (-69% – 198%)	-53% (-86% – 60%)	-68% (-92% – 21%)	-67% (-92% – 39%)	**-88% (-97% – -43%)†**	-38% (-80% – 93%)
	Microscopically confirmed cases	N/A	N/A	N/A	N/A	N/A	#N/A	#N/A	#N/A	#N/A	#N/A	#N/A
	Slid positivity rate	N/A	N/A	N/A	N/A	N/A	#N/A	#N/A	#N/A	#N/A	#N/A	#N/A
	Malaria admissions	14,548	8,863	4,757	-67	-64	-7% (-46% – 59%)	**-62% (-79% – -32%)†**	**-74% (-86% – -49%)†**	**-60% (-80% – -18%)†**	**-82% (-92% – -61%)†**	**-51% (-71% – -16%)†**
	Malaria deaths	540	237	135	-75	-127	-17% (-57% – 62%)	**-64% (-83% – -24%)†**	**-72% (-88% – -37%)†**	**-61% (-84% – -6%)†**	**-77% (-91% – -40%)†**	**-57% (-78% – -17%)†**
**Respiratory infections**	OPD Respiratory infection	925	2,149	2,140	131	57	57% (-53% – 425%)	51% (-60% – 465%)	-14% (-79% – 259%)	12% (-76% – 422%)	-20% (-85% – 321%)	61% (-52% – 439%)
	IPD Respiratory infection	2,600	5,389	7,294	181	52	48% (-7% – 136%)	30% (-23% – 118%)	73% (-2% – 205%)	147% (32% – 359%)	147% (26% – 385%)	98% (24% – 216%)
	Deaths Respiratory infection	152	164	184	21	7	27% (-35% – 149%)	13% (-46% – 138%)	84% (-19% – 316%)	116% (-12% – 427%)	131% (-12% – 509%)	40% (-28% – 174%)
**Diarrhoeal diseases**	OPD Diarrhoeal diseases	1,364	2,705	2,370	74	50	11% (-49% – 141%)	-10% (-62% – 113%)	-50% (-81% – 28%)	-39% (-78% – 72%)	**-69% (-90% – -4%)†**	12% (-48% – 144%)
	IPD Diarrhoeal diseases	2,738	4,178	4,602	68	34	37% (-13% – 116%)	1% (-39% – 67%)	-2% (-44% – 71%)	15% (-37% – 111%)	6% (-45% – 104%)	29% (-18% – 103%)
	Deaths Diarrhoeal diseases	67	73	62	-7	9	56% (-40% – 303%)	18% (-59% – 240%)	54% (-52% – 390%)	51% (-57% – 434%)	35% (-66% – 432%)	28% (-50% – 232%)
**Non-malaria**	Non-malariaOPDcases	20,077	38,628	46,851	133	48	19% (-42% – 143%)	21% (-45% – 167%)	-9% (-62% – 115%)	30% (-49% – 232%)	13% (-59% – 210%)	62% (-21% – 232%)
	Non-malariaIPDcases	18,299	21,362	22,779	24	14	21% (-22% – 88%)	-23% (-52% – 25%)	-38% (-63% – 6%)	-31% (-61% – 23%)	-30% (-62% – 31%)	-6% (-39% – 46%)
	Non-malariaDeaths	1,095	923	938	-14	-19	18% (-41% – 135%)	-5% (-56% – 103%)	-17% (-64% – 93%)	-22% (-69% – 94%)	2% (-62% – 175%)	-9% (-54% – 80%)
**5 AND ABOVE YEARS**												
**All-cause outpatient**	All-cause outpatient cases	115,282	266,230	349,565	203	57	19% (5% – 35%)	4% (-9% – 20%)	**-20% (-31% – -7%)†**	-1% (-16% – 17%)	-13% (-27% – 4%)	48% (31% – 68%)
	All-cause admissions	82,258	109,116	117,285	43	25	28% (-18% – 101%)	11% (-32% – 82%)	-22% (-55% – 34%)	-20% (-55% – 45%)	-10% (-53% – 69%)	12% (-29% – 75%)
	All-cause deaths	2,613	2,723	2,837	9	4	16% (-20% – 68%)	9% (-28% – 65%)	-26% (-53% – 16%)	-16% (-49% – 38%)	-5% (-45% – 64%)	-1% (-32% – 44%)
**Malaria**	Outpatient malaria cases	29,476	6,797	6,711	-77	-334	-75% (-71% – 129%)	-74% (-81% – 86%)	**-72% (-94% – -31%)†**	**-70% (-97% – -52%)†**	-69% (-93% – 37%)	-75% (-86% – 7%)
	Microscopically confirmed cases	N/A	N/A	N/A	N/A	N/A	#N/A	#N/A	#N/A	#N/A	#N/A	#N/A
	Slid positivity rate	N/A	N/A	N/A	N/A	N/A	#N/A	#N/A	#N/A	#N/A	#N/A	#N/A
	Malaria admissions	18,344	10,537	6,654	-64	-74	-66% (-34% – 54%)	**-67% (-68% – -19%)†**	**-67% (-82% – -48%)†**	**-68% (-73% – -14%)†**	**-69% (-83% – -42%)†**	**-66% (-64% – -17%)†**
	Malaria deaths	680	506	411	-40	-34	**-29% (-43% – -7%)†**	-24% (-45% – 171%)	-20% (-73% – 53%)	-15% (-76% – 55%)	**-10% (-67% – -7%)†**	**-29% (-60% – -7%)†**
**Respiratory infections**	OPD Respiratory infection	2,315	3,469	3,956	71	33	24% (-47% – 191%)	6% (-62% – 149%)	-8% (-78% – 73%)	-21% (-79% – 94%)	-32% (-80% – 128%)	24% (-54% – 155%)
	IPD Respiratory infection	2,368	3,228	3,702	56	27	65% (-5% – 99%)	68% (1% – 132%)	71% (-15% – 113%)	74% (-13% – 138%)	78% (3% – 206%)	65% (-1% – 108%)
	Deaths Respiratory infection	155	185	204	31	16	40% (-15% – 64%)	43% (10% – 130%)	47% (-23% – 72%)	50% (-22% – 87%)	54% (-4% – 147%)	40% (-9% – 77%)
**Diarrhoeal diseases**	OPD Diarrhoeal diseases	3,988	2,848	2,421	-39	-40	-50% (-95% – 890%)	-55% (-97% – 941%)	-60% (-99% – 648%)	-64% (-98% – 1,442%)	-67% (-99% – 1,199%)	-50% (-96% – 718%)
	IPD Diarrhoeal diseases	1,813	2,158	2,118	17	16	10% (-37% – 150%)	7% (-41% – 173%)	5% (-61% – 107%)	2% (-62% – 133%)	-1% (-63% – 166%)	10% (-44% – 125%)
	Deaths Diarrhoeal diseases	55	47	49	-12	-18	0% (-53% – 50%)	5% (-33% – 140%)	10% (-55% – 84%)	15% (-50% – 132%)	20% (-47% – 175%)	0% (-46% – 72%)
**Non-malaria**	Non-malariaOPDcases	112,967	262,761	345,609	206	57	104% (-42% – 132%)	49% (-57% – 101%)	8% (-71% – 54%)	-21% (-70% – 84%)	-42% (-78% – 54%)	104% (-24% – 206%)
	Non-malariaIPDcases	63,914	98,579	110,631	73	35	41% (-18% – 121%)	29% (-27% – 115%)	19% (-51% – 59%)	9% (-55% – 64%)	0% (-50% – 99%)	41% (-23% – 106%)
	Non-malariaDeaths	1,934	2,217	2,426	25	13	12% (-25% – 75%)	6% (-34% – 66%)	1% (-55% – 23%)	-4% (-54% – 38%)	-8% (-49% – 67%)	12% (-33% – 55%)
**ALL AGES**												
**All-cause outpatient**	All-cause outpatient cases	136,284	307,007	398,556	192	56	18% (5% – 32%)	3% (-9% – 17%)	-21% (-31% – -10%)†	-2% (-15% – 14%)	-15% (-27% – 0%)	45% (30% – 63%)
	All-cause admissions	115,105	139,342	144,821	26	17	22% (-24% – 98%)	-4% (-44% – 63%)	-32% (-62% – 23%)	-27% (-61% – 38%)	-24% (-61% – 52%)	1% (-38% – 63%)
	All-cause deaths	4,248	3,883	3,910	-8	-9	12% (-33% – 88%)	-4% (-46% – 72%)	-30% (-63% – 32%)	-23% (-61% – 54%)	-12% (-59% – 86%)	-10% (-46% – 51%)
**Malaria**	Outpatient malaria cases	33,198	14,969	9,004	-73	-122	-12% (-69% – 149%)	-41% (-81% – 85%)	-71% (-92% – 2%)	-70% (-92% – 15%)	-71% (-93% – 25%)	-54% (-83% – 31%)
	Microscopically confirmed cases	32,420	11,099	8,528	-74	-192	-17% (-63% – 87%)	**-80% (-92% – -50%)†**	**-88% (-95% – -67%)†**	**-72% (-90% – -19%)†**	**-72% (-91% – -12%)†**	**-67% (-85% – -25%)†**
	Slid positivity rate	35	11	9	-73	-218	**-29% (-39% – -17%)†**	**-73% (-77% – -68%)†**	**-74% (-78% – -68%)†**	**-54% (-62% – -44%)†**	**-58% (-66% – -47%)†**	**-62% (-68% – -56%)†**
	Malaria admissions	32,892	19,401	11,411	-65	-70	-3% (-42% – 62%)	**-56% (-75% – -22%)†**	**-71% (-85% – -46%)†**	**-55% (-77% – -11%)†**	**-76% (-88% – -49%)†**	**-48% (-69% – -14%)†**
	Malaria deaths	1,220	743	546	-55	-64	**1% (-50% – -10%)†**	-21% (-64% – 72%)	-55% (-81% – 7%)	-51% (-81% – 26%)	**-47% (-81% – -10%)†**	**-37% (-69% – -10%)†**
**Respiratory infections**	OPD Respiratory infection	3,241	5,618	6,096	88	42	33% (-49% – 251%)	13% (-61% – 227%)	-31% (-78% – 119%)	-22% (-78% – 173%)	-28% (-81% – 176%)	24% (-53% – 226%)
	IPD Respiratory infection	4,968	8,617	10,996	121	42	43% (-6% – 116%)	40% (-12% – 123%)	56% (-6% – 161%)	104% (16% – 256%)	119% (19% – 302%)	73% (14% – 162%)
	Deaths Respiratory infection	307	349	388	26	12	22% (-27% – 105%)	40% (-21% – 148%)	45% (-23% – 171%)	61% (-19% – 218%)	88% (-10% – 293%)	33% (-20% – 123%)
**Diarrhoeal diseases**	OPD Diarrhoeal diseases	5,352	5,553	4,791	-10	4	-18% (-90% – 580%)	-32% (-93% – 602%)	-61% (-97% – 400%)	-43% (-96% – 798%)	-66% (-98% – 564%)	-26% (-91% – 517%)
	IPD Diarrhoeal diseases	4,550	6,336	6,720	48	28	33% (-26% – 138%)	11% (-42% – 112%)	-4% (-53% – 95%)	9% (-50% – 136%)	5% (-54% – 142%)	23% (-32% – 120%)
	Deaths Diarrhoeal diseases	122	120	111	-9	-2	21% (-38% – 136%)	21% (-42% – 151%)	23% (-45% – 174%)	29% (-46% – 208%)	26% (-51% – 223%)	13% (-42% – 119%)
**Non-malaria**	Non-malariaOPDcases	133,044	301,389	392,460	195	56	17% (-35% – 109%)	-3% (-49% – 85%)	-29% (-65% – 42%)	-18% (-62% – 75%)	-35% (-71% – 48%)	54% (-14% – 176%)
	Non-malariaIPDcases	82,213	119,941	133,410	62	31	32% (-21% – 119%)	14% (-35% – 100%)	-18% (-55% – 51%)	-18% (-58% – 60%)	-7% (-55% – 90%)	19% (-29% – 97%)
	Non-malariaDeaths	3,029	3,140	3,364	11	4	16% (-32% – 100%)	2% (-44% – 86%)	-22% (-60% – 50%)	-19% (-61% – 65%)	-3% (-55% – 111%)	-1% (-43% – 70%)

Note:

[a] Main measure of changes estimated for the midpoint year of 2006–2010 (predicted for mid point of 2006-2010 versus average observed value for 2006-2010)

A positive percentage (ratio between observed and predicted indicators level multiplied by 100) indicates an increase in the indicator.

A negative percentage indicate a decrease of the indicator.

†: Confidence Interval (CI) does not include zero and change of trend (pre versus post-intervention) is statistically significant, (P<0.05)
